# Non-invasive prenatal testing can detect silent cancers in expecting mothers

**DOI:** 10.1016/j.gendis.2023.04.008

**Published:** 2023-05-18

**Authors:** Alessandro Ottaiano, Monica Ianniello, Nadia Petrillo, Mariachiara Santorsola, Luigia De Falco, Salvatore Giovanni Castaldi, Maria Antonietta Castaldi, Valentina Giudice, Carmine Selleri, Giovanni Savarese

**Affiliations:** a Unit of Innovative Therapies for Abdominal Metastases, Istituto Nazionale Tumori di Napoli, IRCCS “G. Pascale”, Via Mariano Semmola, Napoli 80131, Italy; b AMES, Centro Polidiagnostico Strumentale SRL, Via Padre Carmine Fico 24, Casalnuovo Di Napoli 80013, Italy; c Specialization School of Clinical Pathology, Università degli Studi di Salerno, Via Giovanni Paolo II 132, Fisciano 84084, Italy; d Unit of High-Risk Pregnancy and Prenatal Diagnosis, University Hospital “San Giovanni di Dio e Ruggi d'Aragona”, Via San Leonardo, Salerno 84125, Italy; e Hematology and Transplant Center, University Hospital “San Giovanni di Dio e Ruggi d'Aragona”, Via San Leonardo, Salerno 84125, Italy; f Department of Medicine, Surgery and Dentistry, “Scuola Medica Salernitana”, Via Salvador Allende 43, Baronissi 84081, Italy

Pregnancy is a unique physiological state in which several changes occur. One of the most important aspects is the maternal immune system, which sets to tolerate the presence of the developing fetus (a semi-allogeneic organism), while at the same time providing protection against pathogens.^1^ Pregnancy has long been known to affect the risk of certain cancers including breast cancer in the short term, gestational trophoblastic disease (GTD), cervical cancer, and melanoma.^2^^,^^3^ The reasons for the increased risk of these cancers during pregnancy are not fully understood, but several hypotheses have been proposed as the hormonal and immune system changes. Non-invasive prenatal testing (NIPT) offers a safer and more accurate alternative to amniocentesis and chorionic villus sampling (CVS) for screening against chromosomal abnormality associated with severe malformations and neurological alterations [American College of Obstetricians and Gynecologists, and the International Society for Prenatal Diagnosis].^4^ However, this is a complex and delicate issue, and structured recommendations, in this context, have proven to be inefficient since the decision to undergo prenatal testing is frequently a personal one.

NIPT is highly accurate and has a low false-positive rate. Invasive diagnostic testing (amniocentesis or CVS) is typically recommended to confirm the diagnosis after positivity to NIPT. The methodology of NIPT testing involves isolating and analyzing cell-free fetal DNA (cffDNA) from the maternal blood sample. Fetal DNA is detectable in maternal circulation as early as the fifth week of pregnancy and its concentration varies throughout gestation. At NIPT testing, in general, the percentage of fetal DNA in the maternal blood sample ranges from 3% to 13%. Despite implementing measures to minimize the risk of maternal contamination,^5^ it may still occur, which can affect the accuracy of the test. Can a potential source of error be reframed as a resource? Specifically, in cases where a positive NIPT result is accompanied by a negative amniocentesis or CVS, could this indicate the early onset of cancer in the mother?

When the aforementioned scenario arose, we recommended immediate postpartum follow-up. This approach was based on the hypothesis that the positive NIPT result could be indicative of an unknown primary cancer that was silently developing during the pregnancy. Methods for performing NIPT and follow-up are reported in File S1.

Between 2018 and 2022, a total of 100,685 pregnant women aged 18 years or older were analyzed, with a median age of 34.6 years (range: 18–50 years). Of these, 1502 tested positive for NIPT. Among these women, 27 presented with single or multiple aneuploidies. A descriptive flowchart of test results is reported in Figure S1. However, subsequent direct tests on amniotic fluid or placenta did not confirm the presence of genetic alterations. The reasons for genetic testing and the development of tumors are described in Table 1. Examples of NIPT results are shown in Figure S2. Of the women analyzed, 16 developed benign or malignant tumors. Four women did not develop cancer until the writing of this report, and 7 were lost to follow-up. Finally, 23 of the pregnant women successfully carried their pregnancies to term. Ten women refused to undergo the proposed post-partum monitoring. Thus, in 11 cases out of 100,685 women, the positivity of the NIPT test in the absence of other diagnostic indications and the application of clinical-radiological monitoring allowed for early identification and treatment of cancer with only surgery or radiotherapy (plus rituximab in some NHL cases) (Table 1). Treating cancer during pregnancy presents a challenge, as the oncologist must strive to optimize and personalize therapeutic options to ensure the well-being of both the mother and the unborn child to the fullest extent possible. However, it is important to emphasize that in our case series, except for one case, the mothers showed no clinical evidence of cancer during pregnancy. Unfortunately, in this single case, a neoplasm (a soft tissue sarcoma) was clinically detected during the fourth month of pregnancy and was found to be highly aggressive. The mother declined the termination of pregnancy to undergo therapy. Following the birth of a perfectly healthy child, the cancer had metastasized to multiple distant sites. In another case, the patient died from oncological problems, having refused to undergo regular check-ups, and the neoplasm was discovered only when clinically symptomatic. In four women, benign lesions such as uterine myomas or breast fibroadenomas were discovered. In these cases, it is possible that these lesions, due to their altered genetics, may have represented pre-cancerous events that were promptly removed.

**Table 1:** Follow-up information for women who tested positive on NIPT but negative on direct diagnostic tests.

Patient initials	Age	Indication for NIPT	Date of blood draw	Type of tumor	Type of aneuploidy	Adherence to post-partum follow-up	Full-term pregnancy	Cancer stage	Type of treatment for cancer	Vital status
AR	34	PC	May 4, 2021	None	Multiple	Yes	Yes	NA	None	A, NED
AV	29	PC	November 6, 2019	FTC	Multiple	Yes	Yes	Stage I	Surgery	A, NED
BMV	39	MAP	June 15, 2021	Unknown^a^	Multiple	No	Yes	Unknown	Unknown	LFU
CMP	31	PC	October 14, 2021	Uterin myoma	Multiple	Yes	Yes	NA	Surgery	A, NED
CS	39	Age	March 3, 2021	Uterin myoma	Multiple	Yes	Yes	NA	Surgery	A, NED
Ca Ma	42	MAP	January 8, 2022	Unknown^a^	Multiple	No	Yes	Unknown	Unknown	LFU
Cr Ma	38	Age	December 19, 2018	None	Multiple	Yes	Yes	NA	None	A, NED
CD	30	PC	February 4, 2022	Unknown^a^	Multiple	No	Yes	Unknown	Unknown	LFU
EE	36	Age	December 28, 2020	STS	Single (13)	No	Yes	Stage IV	CT	D (May 6, 2022) progression of STS
Fa Va	26	PC	June 14, 2021	NHL	Multiple	Yes	Yes	Stage II	Rituximab + RT	A, NED
Fe Va	28	PC	December 6, 2018	NHL	Multiple	Yes	Yes	Stage I	RT	A, NED
FF	37	Age	April 12, 2022	SFT of SNC	Multiple	Yes	Yes	Diameter 2 cm, no metastases	Surgery	A, NED
FL	39	Age	April 26, 2022	Unknown^a^	Multiple	No	Yes	Unknown	Unknown	LFU
FR	35	Age	June 16, 2021	None	Multiple	Yes	Yes	None	None	A, NED
LB	37	Age	September 25, 2021	BC	Multiple	Yes	No	Stage I	Surgery + HT	A, NED
LR	35	Age	November 29, 2021	Breast fibroadenoma	Multiple	Yes	Yes	NA	Surgery	A, NED
Mar Car	39	Age	September 8, 2022	NHL	Multiple	Yes	Yes	Stage II	Rituximab + RT	A, NED
Mas Car	35	MAP	August 10, 2021	Unknown^a^	Multiple	No	Unknown	Unknown	Unknown	LFU
PA	40	Previous T21	January 25, 2020	Colon	Multiple	No	Yes	Stage II	Surgery	A, NED
PF	34	PC	December 22, 2021	NHL	Multiple	Yes	Yes	Stage II	RT	A, NED
PMR	33	MAP	May 18, 2021	None	Multiple	Yes	Yes	NA	None	A, NED
PJ	28	PC	June 29, 2021	BC	Multiple	No	Yes	Stage I	Surgery + HT	D (Sept 22, 2022) progression of BC
PE	40	Age	August 25, 2021	Unknown^a^	Multiple	No	No	Unknown	Unknown	LFU
TG	45	MAP	January 7, 2022	Breast fibroadenoma	Multiple	Yes	Yes	NA	Surgery	A, NED
TP	35	Age	January 26, 2021	Unknown^a^	Multiple	No	Unknown	Unknown	Unknown	LFU
VMC	23	PC	February 19, 2021	NHL	Multiple	Yes	Yes	Stage I	RT	A, NED
WN	26	PC	September 6, 2019	Colon	Multiple	Yes	Yes	Stage II	Surgery	A, NED

A, alive; BC, breast cancer; CT, chemotherapy; D, dead; FTC, follicular thyroid carcinoma; HT, hormone therapy; LFU, lost to follow-up; MAP, medically-assisted procreation; NED, no evidence of disease; NHL, non-Hodgkin lymphoma; PC, personal choice; RT, radiotherapy; SFT, solitary fibrous tumor; STS, soft tissue sarcoma.

a These patients did not want to undergo any further diagnostic investigations or release any additional information. Indirect information obtained through the treating physician suggests a high probability that they may have developed cancer.

The first direct and intuitive consideration is that 11 cases out of 100,685 are an extremely small fraction to attribute relevance to NIPT. However, in Italy and most European countries, millions of pregnant women undergo this procedure each year, either by personal choice or because they belong to one of the high-risk categories. This means that hundreds of similar situations could be identified each year.

Four women presented with a positive NIPT test result, without any other diagnostic evidence (amniocentesis and CVS) of chromosomal aberrations, and gave birth to a completely normal newborn and did not develop cancer. However, the immediate repetition of these tests has resulted in positive NIPT once again. In these cases, anxiety and a significant cost to the healthcare system can be triggered, as these patients have undergone unnecessary checks. Genetic mosaicism cannot be ruled out; however, these positive tests could be related to a dynamic and transitory phenomenon. It could be the result of contamination by tumor DNA during a phase of elimination of tumor cells by the immune system (“immunological surveillance”). This report does not provide specific experimental data on this issue. However, we can leverage the advantage of having a biobank that preserves plasma samples from the women included in this study. We are using this resource to explore these cases in detail aiming to distinguish fetal DNA from circulating tumor DNA with greater precision (a patent currently under development cannot be disclosed) and to assess the presence of an anti-tumor immune response. The study is ongoing.

There are many ethical questions, and the subject matter is delicate and rich in opportunities for reflection and repercussions in terms of psychology and privacy issues. In fact, from a psychological point of view, in our experience, in some cases, communicating a positive NIPT result alongside the negativity of other diagnostic tests has generated a strong reaction of flight and anger, especially in women in a lower socioeconomic and cultural context. Unfortunately, we do not have the authorization to provide information on clinical events following delivery for these patients. Genetic counseling in this scenario should be integrated with psychological counseling to manage patients' reactions.

We recommend that all women with a positive NIPT test, in the absence of other relevant diagnostic indications from amniocentesis or CVS, undergo strict clinical-radiological monitoring after delivery to exclude the possibility of developing cancer. A methodologic limitation of our study is the absence of a follow-up of at least 5 years for all women diagnosed with cancer, which would allow us to declare them cured with greater certainty. However, we believe that further studies are urgently warranted to shed light on the role of NIPT in the detection of silent cancers in expecting mothers since, at this stage, the likelihood of successful treatment is considerably high.

## Author contributions

Conceptualization, A.O., M.I.; methodology, N.P., M.S., L.D.F.; software, A. O, C.S., G.S.; validation, A.O., C.S., G.S.; investigation, M.I., S.G.C., M.A.C., V.G.; resources, G.S.; data curation, A.O., N.P., M.S.; writing—original draft preparation, A.O., M.S., G.S.; writing—review and editing, N.P., V.G., C.S. All authors read and agreed to the published version of the manuscript.

## Conflict of interests

All authors declare that there is no conflict of interests related to this manuscript.

## Funding

This work was supported by grants from the Centro AMES.

## Data availability

Genomic sequencing results of deidentified participants included in Figure S2 are available at https://zenodo.org/record/7849442#.ZEGnp_zP25c.
