# Supplementary material for: Non-invasive prenatal testing can detect silent cancers in expecting mothers
Source: Genes Dis. 2023 May 18;11(2):585–8. doi: 10.1016/j.gendis.2023.04.008 (PMC10491905; doi:10.1016/j.gendis.2023.04.008)
Supplement: Multimedia component 1 [file mmc1.docx]

**Non-invasive prenatal testing can detect silent cancers in expecting mothers.**

**Patients and methods**

**Patients’ selection, clinical information, and counselling timing**

The study participants were nationwide pregnant women aged 18 years or older who were referred to our laboratory for genetic testing. The laboratory holds accreditation for prenatal and genetic disease testing according to UNI EN ISO 9001:2008 standards. The study included low-risk women who chose NIPT as a first-tier test, as well as women considered to be at high risk for fetal aneuploidies. High-risk criteria included maternal age of 35 years or older, previous pregnancy with aneuploidy, high-risk first trimester combined test results, abnormal ultrasound findings (including nuchal translucency of over 3.5 mm), parents with balanced chromosomal abnormalities or other chromosomal rearrangements, or a family history of aneuploidy. However, patients with maternal age over 45 years, abnormal ultrasound findings, or family history of genetic disorders were advised to undergo diagnostic tests such as CVS or amniocentesis. The cohort included singleton, monochorionic, and dichorionic twin pregnancies, as well as naturally conceived or medically-assisted procreation (MAP) pregnancies. Chorionicity was assessed by ultrasound, and patients were accepted from 9 weeks gestation onwards. Pre-test counselling was provided to all patients, and written informed consent was obtained before blood collection. Data were prospectively collected from the patients, including their medical history, age, and pregnancy status. The blood samples were processed in our

laboratory, and fetal DNA was analyzed to determine the likelihood of the fetus having a chromosomal abnormality. Interpretation of the results was based on the analysis of chromosomes. In the case of an abnormal test result, additional counselling was provided by a clinical geneticist, and confirmatory diagnostic testing using material obtained via amniocentesis or CVS was offered.

In this study, patients with positive NIPT results and negative diagnostic testing were invited for further optional diagnostic investigations after delivery, including total-body computed tomography (CT) scan within 3 months, senologic, gynecologic, and dermatologic specialist visits. In any case, patients were followed up through regular visits to their referral gynecologist and family doctor. Stages of cancer diagnosed during this study have been defined according to TNM classification of malignant tumours, updated editions. Anti-cancer treatments were selected based on the latest AIOM (Associazione Italiana di Oncologia Medica) guidelines and in accordance with the decisions and expectations of the patients, considering the delicate psychological context of their lives.

**Non-invasive pre-natal testing (NIPT)**

Peripheral blood samples (10 mL) were collected from pregnant women during the first trimester of pregnancy. The blood was collected in special tubes that prevent clotting and ensure the stability of the DNA (STRECK Cell-Free DNA BCT, Streck Corporate, NE 68128, USA). The samples were either collected at our laboratory or sent there from other laboratories within Italy. The latter samples were sent at a controlled temperature of 4 °C, and the maximum interval between blood draw and arrival at our center was 5 days. Samples were centrifuged at 1600 rcf (relative centrifugal force), 4°C for 10 minutes, to separate plasma from peripheral blood. Then, 900 μL of the supernatant were transferred to a new deep well plate for an additional 10 minute centrifugation at 5600 rcf (same conditions mentioned before) and used for cell-free fetal DNA (cffDNA) extraction. Briefy, cffDNA extraction and purification were achieved by adsorption onto a binding plate; the binding plate was washed to remove contaminants, followed by eluting. The pipeline included an automated library preparation (VeriSeq NIPT Solution v1 and v2, Microlab STAR, assay) followed by whole genome sequencing (WGS) on NextSeq550 and NextSeq 550Dx, respectively (Illumina Inc., San Diego, CA, USA).

**Biostatistics pipeline**

The VeriSeqNIPT Assay Software versions 1 and 2 (www.illumina.com/NIPTsoftware) were used to analyze the aneuploidy status, fetal fraction, common trisomies, and sex chromosomes (SCA, Sexual Chromosome Aneuploidies), as well as rare autosomal aneuploidies and partial deletions/duplications ≥ 7 Mb (**1-4**). The generated WGS data were streamed to the VeriSeq NIPT Analysis Server, where the software filtered and aligned the WGS reads to a human reference genome. Software uses a counting‐based algorithm to generate the log-likelihood ratio (LLR) scores for chromosomes 13, 18, and 21, as well as NCV_X and NCV_Y scores for sex classification for each sample. LLR thresholds for calling a sample high or low risk were internally validated, and a decision tree was agreed upon for handling failures (**1**). Samples failed when the sequencing coverage was judged insufficient based on the fetal fraction estimate for the sample, as indicated by the Individualized Fetal Fraction Confidence Test (iFACT), which is a quality control parameter of the VeriSeqTM NIPT Solution v1. If samples repeatedly failed due to data outside of the expected range (DOER), a genome-wide data analysis was performed with an in-house developed algorithm to identify whether rare aneuploidy was the cause of repeated failure (**2, 3**). Resampling and reanalysis were performed at no extra cost. Singleton and multiple gestation pregnancy samples were handled in the same way. The statistical analysis of the data was conducted utilizing the statistical set SPSS for Windows (version 20 SPSS Inc., Chicago, IL).

**References**

1. La Verde M, De Falco L, Torella A, Savarese G, Savarese P, Ruggiero R, Conte A, Fico V, Torella M, Fico A. Performance of cell-free DNA sequencing-based non-invasive prenatal testing: experience on 36,456 singleton and multiple pregnancies. BMC Med Genomics. 2021;30;14(1):93. doi: 10.1186/s12920-021-00941-y.
2. Bayindir B, Dehaspe L, Brison N, Brady P, Ardui S, Kammoun M, Van der Veken L, Lichtenbelt K, Van den Bogaert K, Van Houdt J, Peeters H, Van Esch H, de Ravel T, Legius E, Devriendt K, Vermeesch JR. Noninvasive prenatal testing using a novel analysis pipeline to screen for all autosomal fetal aneuploidies improves pregnancy management. Eur J Hum Genet. 2015;23(10):1286-93. doi: 10.1038/ejhg.2014.282.
3. Straver R, Sistermans EA, Holstege H, Visser A, Oudejans CB, Reinders MJ. WISECONDOR: detection of fetal aberrations from shallow sequencing maternal plasma based on a within-sample comparison scheme. Nucleic Acids Res. 2014;42(5):e31. doi: 10.1093/nar/gkt992.
4. Pertile MD, Flowers N, Vavrek D, Andrews D, Kalista T, Craig A, Deciu C, Duenwald S, Meier K, Bhatt S. Performance of a Paired-End Sequencing-Based Noninvasive Prenatal Screening Test in the Detection of Genome-Wide Fetal Chromosomal Anomalies. Clin Chem. 2021;67(9):1210-1219. doi: 10.1093/clinchem/hvab067.
