# Supplementary material for: Non-invasive prenatal testing can detect silent cancers in expecting mothers
Source: Genes Dis. 2023 May 18;11(2):585–8. doi: 10.1016/j.gendis.2023.04.008 (PMC10491905; doi:10.1016/j.gendis.2023.04.008)
Supplement: Multimedia component 2 [file mmc2.pptx]

## Slide 1
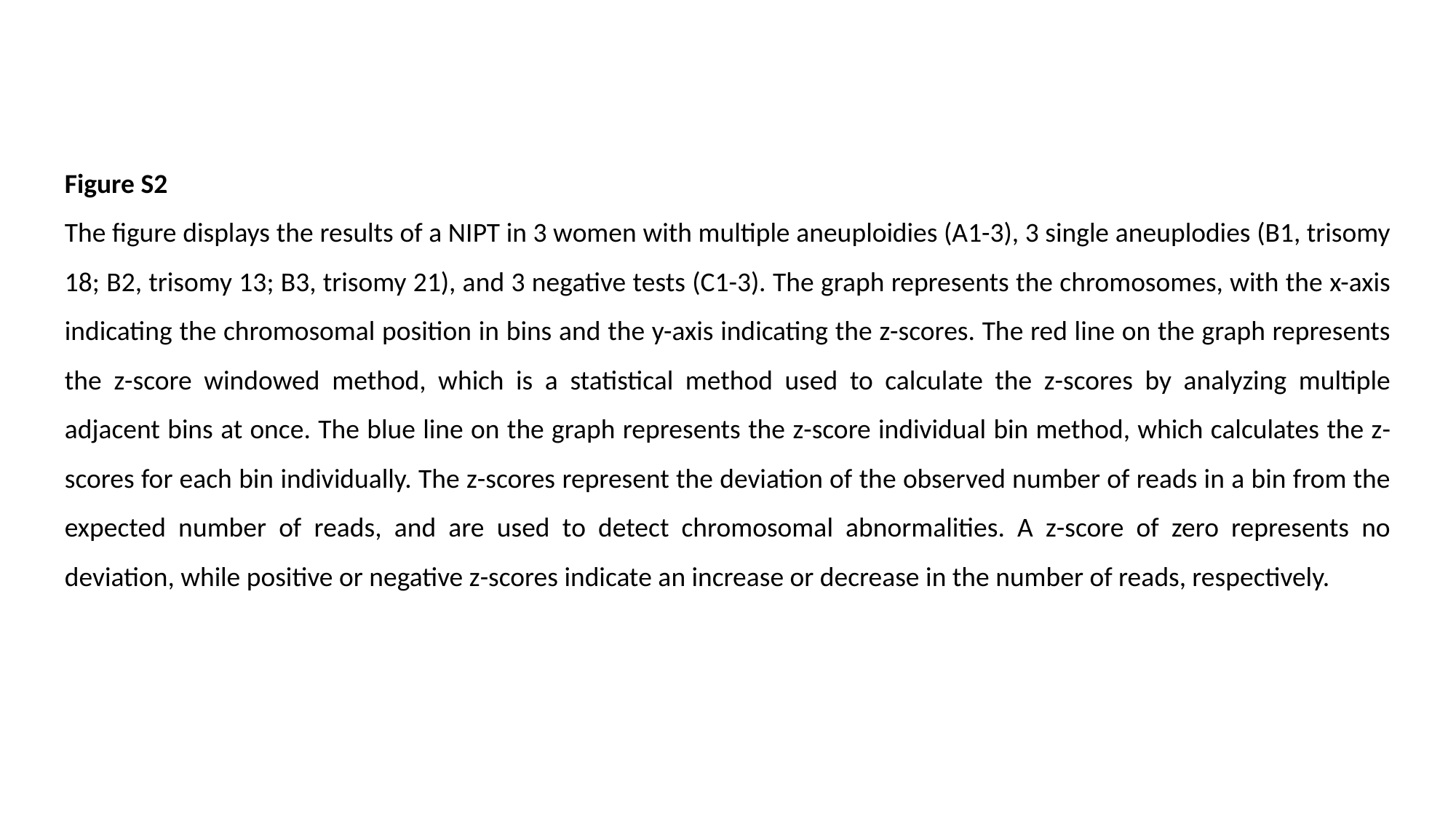

Figure S2
The figure displays the results of a NIPT in 3 women with multiple aneuploidies (A1-3), 3 single aneuplodies (B1, trisomy 18; B2, trisomy 13; B3, trisomy 21), and 3 negative tests (C1-3). The graph represents the chromosomes, with the x-axis indicating the chromosomal position in bins and the y-axis indicating the z-scores. The red line on the graph represents the z-score windowed method, which is a statistical method used to calculate the z-scores by analyzing multiple adjacent bins at once. The blue line on the graph represents the z-score individual bin method, which calculates the z-scores for each bin individually. The z-scores represent the deviation of the observed number of reads in a bin from the expected number of reads, and are used to detect chromosomal abnormalities. A z-score of zero represents no deviation, while positive or negative z-scores indicate an increase or decrease in the number of reads, respectively.

## Slide 2
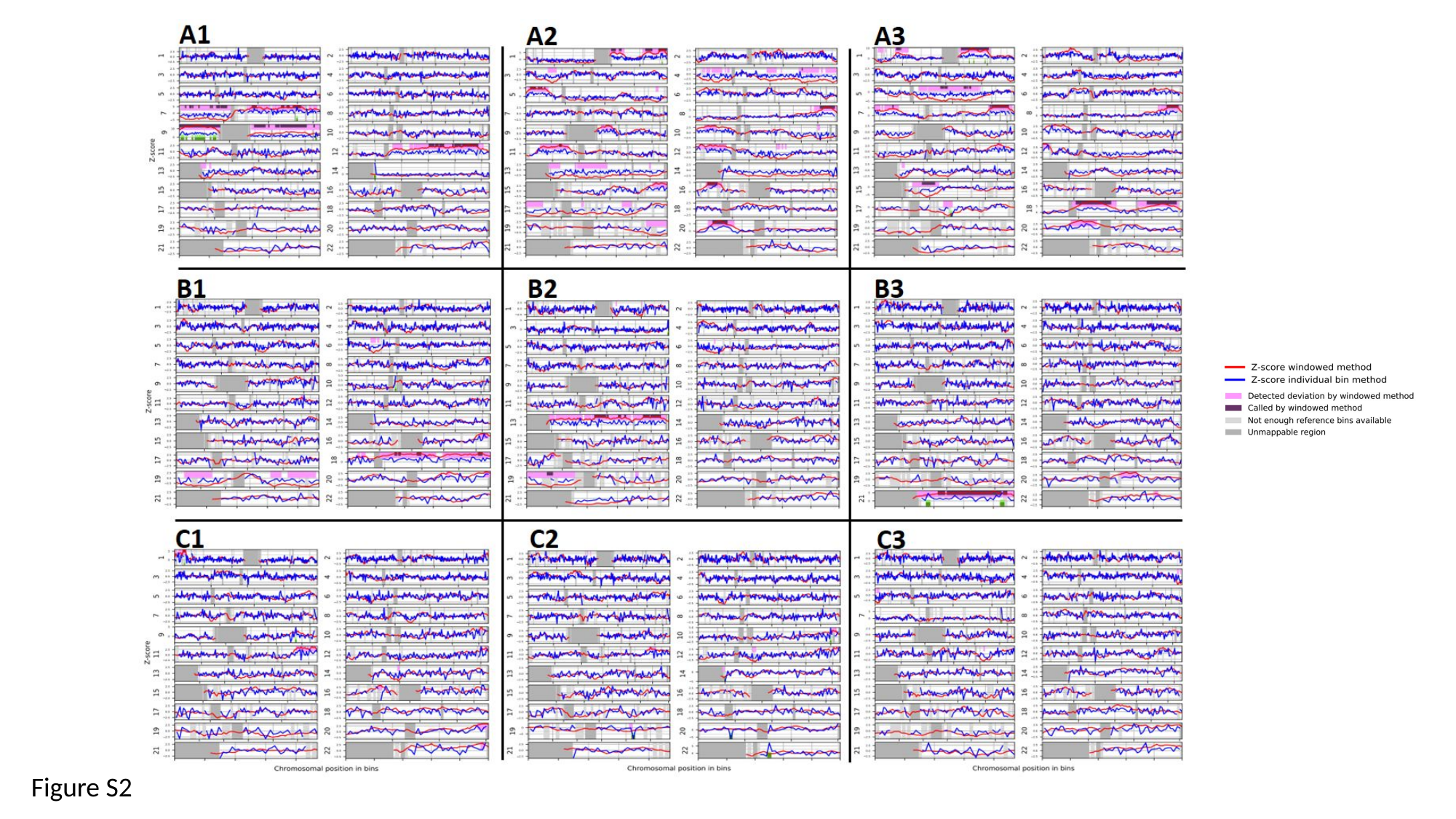

Figure S2
