# Supplementary figures and images for: Non-invasive prenatal testing can detect silent cancers in expecting mothers
Source: Genes Dis. 2023 May 18;11(2):585–8. doi: 10.1016/j.gendis.2023.04.008 (PMC10491905; doi:10.1016/j.gendis.2023.04.008)

## Slide 1
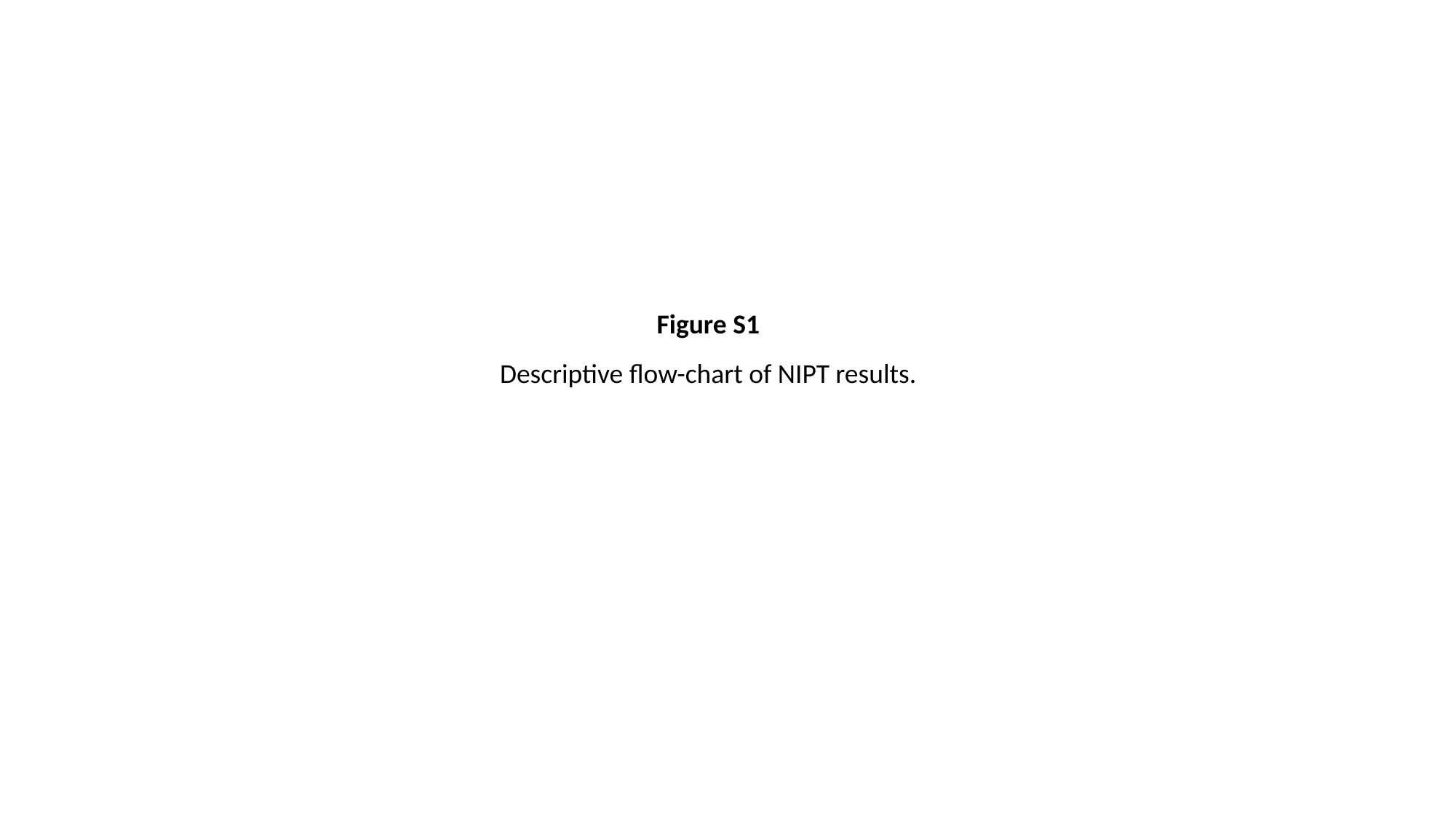

Figure S1
Descriptive flow-chart of NIPT results.

## Slide 2
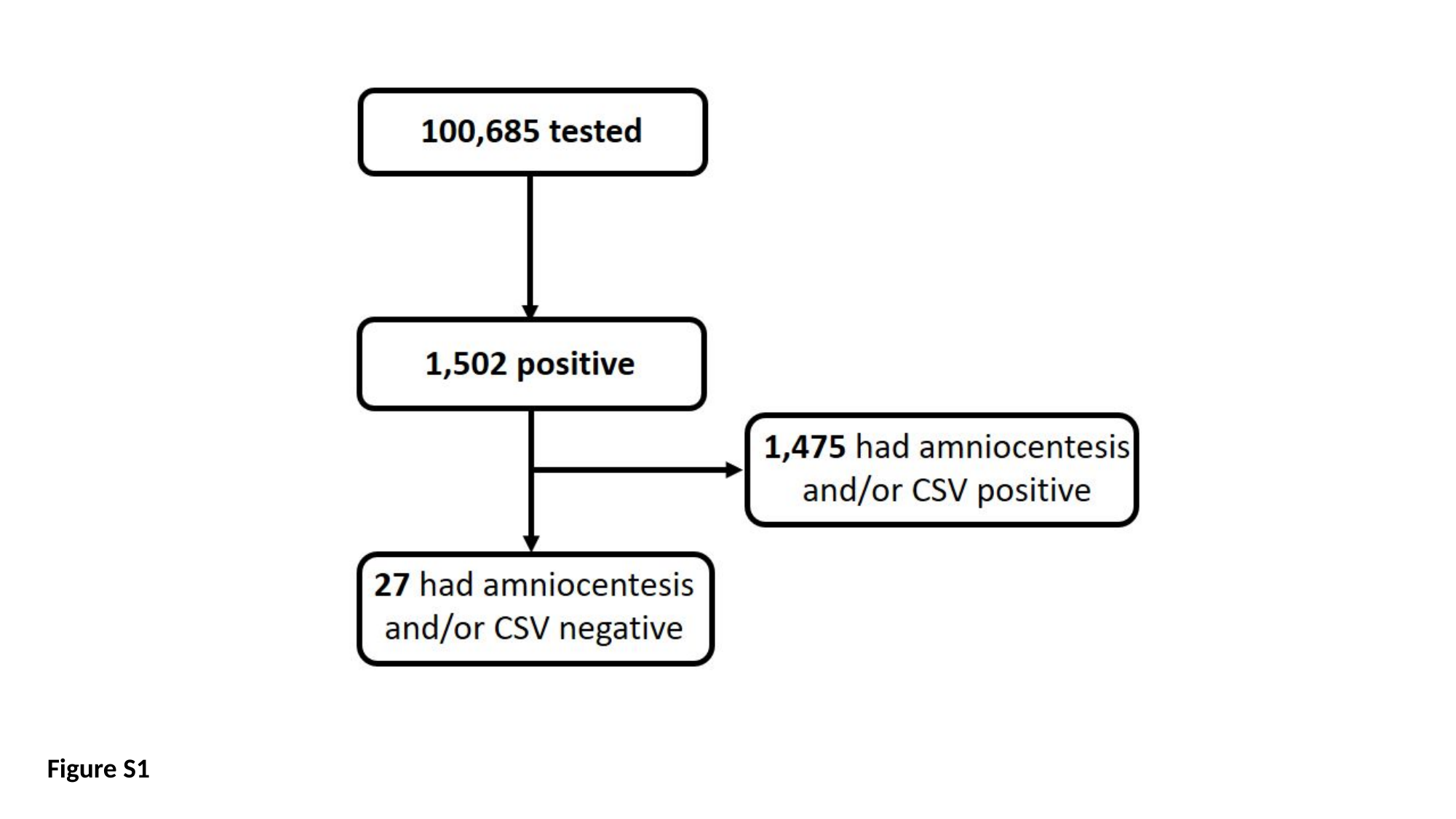

Figure S1

Supplement: Multimedia component 3 [file mmc3.pptx]
